# Income loss and fertility intentions during the COVID-19 pandemic in Brazil*

**DOI:** 10.20947/s0102-3098a0267

**Published:** 2024-12-06

**Authors:** Letícia Junqueira Marteleto, Molly Dondero, Luiz Gustavo Fernandes Sereno, Raquel Zanatta Coutinho

**Affiliations:** ** University of Pennsylvania, Philadelphia, PA, United States.; *** American University, Washington, DC, United States.; **** University of Pennsylvania, Philadelphia, PA, United States.; ***** Federal University of Minas Gerais, Belo Horizonte-MG, Brazil.

**Keywords:** COVID-19, Socioeconomic shocks, Fertility, Reproductive behaviors, Probabilistic survey, COVID-19, Choques socioeconômicos, Fecundidade, Comportamentos reprodutivos, Pesquisa probabilística, COVID-19, Impactos socioeconómicos, Fecundidad, Comportamientos reproductivos, Encuesta probabilística

## Abstract

The study aims to examine how pandemic-induced income loss shaped fertility intentions at the pandemic’s onset, examining differences in this association between mothers and non-mothers, and whether effects are similar for intentions to postpone *versus* forgo childbearing. The research employs a mixed-method approach, combining population-level probabilistic survey data from 1,524 fecund partnered women aged 18–34 with qualitative insights gathered from 56 semi-structured interviews with women aged 18–39 in Pernambuco, Brazil. Multinomial regression models were utilized to distinguish between intentions to postpone, forgo, and get pregnant within six months, exploring associations with pandemic-induced income loss prior to the interview, motherhood status, and parity. We find that most women intended to avoid pregnancy at the onset of the pandemic, with key differences between intentions to postpone *versus* forgo between mothers *versus* non-mothers. Further, pandemic-induced income loss and motherhood/parity interacted to define fertility intentions. Whereas income loss did not affect non-mothers, mothers had different intentions depending on income loss, with those experiencing it more likely to postpone or forgo a pregnancy, and mothers of two or more children more likely to forgo a pregnancy altogether. Qualitative analysis corroborated these patterns and provided further nuanced sensitivity of fertility intentions to pandemic-induced income shocks.

## Resumo

O estudo objetiva examinar como a perda de renda induzida pela pandemia moldou as intenções de fecundidade no início da pandemia, examinando as diferenças nessa associação entre mães e não mães, e se os efeitos são semelhantes para as intenções de adiar *versus* renunciar à gravidez. A pesquisa emprega uma abordagem mista, combinando dados de pesquisa probabilística em nível populacional de 1.524 mulheres férteis com parceiros, com idades entre 18 e 34 anos, com observações qualitativas obtidas a partir de 56 entrevistas semiestruturadas com mulheres com idades entre 18 e 39 anos em Pernambuco, Brasil. Modelos de regressão multinomial foram utilizados para distinguir entre intenções de adiar, renunciar e engravidar dentro de seis meses, explorando associações com a perda de renda induzida pela pandemia antes da entrevista, maternidade e parturição. Observamos que a maioria das mulheres pretendia evitar a gravidez no início da pandemia, com diferenças importantes entre as intenções de adiar *versus* renunciar entre mães e não mães. Além disso, a perda de renda induzida pela pandemia e a maternidade/parturição interagiram para definir as intenções de fecundidade. Enquanto as não mães não foram afetadas pela perda de renda, as mães tinham intenções diferentes dependendo da perda de renda, com aquelas que a experimentaram mais propensas a adiar ou renunciar a uma gravidez, e as mães de dois ou mais filhos mais propensas a renunciar completamente a uma gravidez. A análise qualitativa corroborou esses padrões e forneceu uma sensibilidade mais sutil das intenções de fecundidade aos choques de renda induzidos pela pandemia.

## Resumen

El estudio tiene como objetivo examinar cómo la pérdida de ingresos inducida por la pandemia moldeó las intenciones de fecundidad al comienzo de la pandemia de COVID-19, examinando las diferencias en esta asociación entre madres y no madres, y si los efectos son similares para las intenciones de posponer *versus* renunciar al embarazo. La investigación emplea un enfoque mixto que combina datos de encuestas probabilísticas a nivel poblacional de 1524 mujeres fértiles con parejas, de entre 18 y 34 años, con observaciones cualitativas obtenidas de 56 entrevistas semiestructuradas con mujeres de entre 18 y 39 años en Pernambuco, Brasil. Se utilizaron modelos de regresión multinomial para distinguir entre intenciones de posponer, renunciar y quedar embarazadas en seis meses, explorando asociaciones con la pérdida de ingresos inducida por la pandemia antes de la entrevista, maternidad y paridad. Encontramos que la mayoría de las mujeres tenían la intención de evitar el embarazo al inicio de la pandemia, con diferencias importantes entre las intenciones de posponer *versus* las de renunciar entre madres y no madres. Además, la pérdida de ingresos consecuencia de la pandemia y la maternidad/paridad interactuaron para definir las intenciones de fecundidad. Mientras que la pérdida de ingresos no afectó a las no madres, las madres tenían intenciones diferentes según la pérdida de ingresos, siendo aquellas que la experimentaron más propensas a posponer o renunciar a un embarazo, y las madres de dos o más hijos más propensas a renunciar por completo a un embarazo. El análisis cualitativo corroboró estos patrones y proporcionó una sensibilidad más sutil de las intenciones de fecundidad a los impactos de ingresos inducidos por la pandemia.

## Introduction

Women, particularly mothers of young children, faced a disproportionate share of the negative socioeconomic consequences of the COVID-19 pandemic, such as income loss and increased housework. This uneven burden has led demographers to question whether the demographic consequences of the pandemic extend beyond mortality and morbidity to affect fertility intentions and behaviors (Berrington *et al*., 2021). Increasing evidence, primarily from Asia, the U.S., and Europe, suggests the pandemic contributed to increases in intentions to avoid pregnancy (Kahn *et al*., 2021; Lin *et al*., 2021; Luppi; Arpino; Rosina, 2020; Novelli *et al.*, 2021), that such intentions to avoid pregnancy were higher early on but persisted throughout the pandemic (Lindberd *et al*., 2021), and that financial concerns are a primary factor underlying changes in intentions (Lindberg *et al*., 2021; Malicka; Mynarska; Świderska, 2021).

Fertility intentions are dynamic and often shift in response to macro- and meso-level household shocks, serving as an important barometer of social change in one of the most consequential decisions in many women’s lives – whether and/or when to have (more) children (Rotkirch, 2020). Thus, understanding the nuanced sensitivity of fertility intentions to pandemic-induced income loss can provide insights into the demographic consequences of the pandemic. To date, there is little research about how pandemic-induced socioeconomic shocks shaped fertility intentions, whether the associations between such shocks and fertility intentions differ for mothers *versus* non-mothers, and whether effects are similar for intentions to postpone *versus* permanently forgo pregnancy.

This study examines three research objectives. First, we investigate whether women intended to avoid pregnancy at the onset of the pandemic, either by postponing or by forgoing pregnancy. Second, we examine how income loss early in the pandemic contributed to intentions to avoid pregnancy. Third, we examine whether income loss operated differently for the intentions of mothers *versus* non-mothers. To address these objectives, we combine analyses of a unique population-representative survey and qualitative data from Brazil, an epicenter of the COVID-19 pandemic (WHO, 2021). By the end of 2023, Brazil had reached almost 700,000 confirmed COVID-19 deaths and 37 million cases, second only to the U.S. (Johns Hopkins Coronavirus Resource Center, 2024).

## Literature review

### Public health crisis and impact on fertility intentions

In less than 10 years, Brazil underwent two public health crises: the Zika Virus epidemic and the COVID-19 pandemic. In the case of Zika, the outbreaks of microcephaly in fetuses and newborns, which, together with a myriad of other symptoms, constitute what researchers then called Congenital Zika Syndrome (CZS), is known to have affected reproductive plans and be associated with a decreasing number of live births in Brazil in late 2015 and in 2016 (Castro *et al*., 2018; Marteleto *et al*. 2020; Rangel; Nobles; Hamoudi, 2020). The Northeast region had 76.1% of all confirmed cases in the country, with the State of Pernambuco alone holding 20.1% of them (Brasil, 2024).

Regarding COVID-19, the impact on mortality was striking in the country.^1^ Brazil registered 711,380 deaths during the pandemic, with larger concentrations in the second wave (April 8th, 2021 with a peak of 4190 deaths/day), followed by the first wave (July 29th, 2020, with 1554 deaths/day), and the third wave (February 22nd, 2022, with 1174 deaths/day) (Worldometer, 2024).

The COVID-19 pandemic had the potential to cause even worse reproductive health outcomes than the Zika epidemic, due to major increases in maternal mortality (Souza; Amorim, 2021). In terms of consequences for infants and children, despite the low lethality of the virus for this population group, differences in survival exist between developing and developed countries. Brazil experienced the highest pediatric death rate for this cause, 23 per 1 million children compared to 2 per 1 million in the United States (Kitano *et al*., 2021).

Importantly, the demographic consequences of the pandemic extend beyond mortality and morbidity to affect fertility (Berrington *et al*., 2021), with possible mixed expectations (AASSVE; Le Moglie; Mencarini, 2021; Coutinho *et al*., 2020). On the one hand, researchers expected increases in pregnancies and live births due to limited contraceptive availability – especially for low-income women - and changed job routines, such as remote work – for high-income women. On the other hand, previous research has shown how fear of infection (Trinitapoli; Yeatman, 2018), combined with social turmoil and economic uncertainty (Sobotka; Skirbekk; Philipov, 2011) could increase the desire to postpone or interrupt reproductive plans, resulting in a decrease in fertility intentions and live births. In fact, a recent study has shown that fertility intentions declined during the pandemic in Brazil (Marteleto *et al*., 2023a). That is, the social and economic consequences of the pandemic stretch beyond its health and mortality consequences as the possibility of unemployment or moving to a precarious job and having a reduced standard of living increase uncertainty.

### Fertility intentions

There is a long and expanding literature about the meaning of fertility intentions^2^ as well as their measurement and theoretical implications. Although fertility intentions are correlated with fertility behavior at the aggregate level, at the individual level, there is a discrepancy mostly caused by the timing between them (Morgan, 2001; Bernardi; Mynarska; Rossier, 2014). As intentions are not static, but dynamic, they often shift in response to changes in life course and structural conditions (Rotkirch, 2020).

Varying from “definitively yes – a child is in the plan” to “definitively no – a child is not in the plans”, a qualitative work found six categories that cover a range of situations in which couples form their reproductive intentions or explain timing until the (next) child. This diversity emerges from contextual changes and situations that are out of people’s control, such as obstacles (not being in a union, lack of employment, lack of housing), competing life goals (education or career), or even waiting for emotional readiness (Bernardi; Mynarska; Rossier, 2014). Thus, intentions are generated by what Bhrolchain and Beaujouan (2015) call a constructive process, in response to context-dependent decisions and people’s changeable preferences across the life course. A study that took place in 2019 used a controlled laboratory experiment for heterosexual partners (n = 838) randomly assigned participants to read either a negative or a positive future economic scenario. Compared to the control group who were not assigned any scenario, the group who read a negative scenario presented a clear decrease in fertility intentions, while the group exposed to the positive scenario presented an increase in fertility intentions, irrespective of gender (Lappegard *et al*., 2022).

It is important to note that ambivalence in intentionality is highly prevalent. At any given point of her longitudinal interviews (3 waves lasting 12 months), Jones (2017) found roughly 20% of the sample unsure whether they wanted to have (more) children – but only 9% were uncertain on all three surveys. Uncertainty was associated with being older, unmarried, having a higher number of children ever born, and having a partner who was also unsure (Jones, 2017). In their study, the only group for whom intentions were consistent with behavior were those with negative intentions (Bhrolcháin; Beaujouan, 2015).

The COVID-19 pandemic provides an important framework to understand the perceived influence of the future on the course of action. Guetto, Bazzani, and Vignoli (2022) compared measured individuals’ expectations concerning the duration of the pandemic and fertility intentions, finding that respondents’ perceptions of health and work insecurity were associated with having reduced fertility intentions during the lockdown. Besides, larger risks of declining fertility were found for those who thought that it would take a long time for life to go back to normal. Another interesting finding is that the expected level of happiness from having a(nother) child was positively associated with a higher post-pandemic fertility intention, but the effect is larger for first or second children compared to those who have at least two children, which the authors interpret as a lower perceived gain from higher-order childbirths (Guetto; Bazzani; Vignoli, 2022).

### What models changes in fertility intentions during the COVID-19 pandemic?

Uncertainty is a characteristic of contemporary societies (Guetto; Bazzani; Vignoli, 2022), and, since the pandemic has added a thick layer of instability, it is crucial to analyze its possible effects on intentions and the variables that could mediate the relationship observed. A growing literature on fertility intentions has explored the effects of COVID-19 on fertility intentions according to parity (existing children) and socioeconomic variables, such as education level, occupation characteristics, and income.

Several of those studies took place in Asia. A study in China shows that half of those who planned to have a child changed their fertility plans due to COVID-19. Older individuals and those planning their second child were particularly prone to abandoning their childbearing plans due to COVID-19 (Zhou; Guo, 2023). Other studies found similar results, with 31.5% of respondents in China planning not to have any more children (Chen *et al*., 2022); in Shanghai 30% of couples who were trying to conceive canceled their pregnancy plans after the COVID-19 outbreak (Zhu *et al*., 2020); and 47.7% of couples who had the intention of having a child were affected by the outbreak (Chu *et al*., 2022). In Singapore, a study that compared fertility intentions in both Zika and COVID-19 public health crises found that the Zika outbreak caused smaller delays in intentions (with 7.5% intending to delay childbearing and only 0.6% intending to decline fertility) while COVID-19 led to larger delays (15%) and quantum effects (5%) (Tan; Ryan; Lim-Soh, 2021). They also found a link between the two epidemics, with women who had already delayed childbearing due to Zika being more likely to further adjust the timing of childbearing due to COVID-19 (both anticipating and postponing). While Zika adjustments were made due to fear of infections, COVID-19 adjustments were made due to income loss during the pandemic (Tan; Ryan; Lim-Soh, 2021).

Those results were highly stratified by women’s socioeconomic status. In Chen’s study (2022), increasing income impacts perceived behavioral control, which in turn, improves women’s confidence in having a child. For Zhu *et al*. (2020), participants who believed in the government were less likely to change their intention to become pregnant. As for Tan, Ryan, and Lim-Soh (2021), college-educated women were more likely to intend to delay childbearing during the lockdown. This finding goes hand in hand with another study, this time for South Korea. As time spent at home increases due to remote working, individuals, especially women, tend to delay or forgo fertility, especially if they already have one child, which could be explained by increased housework associated with the lockdown (Kwan; Choi, 2022).

One of the first manuscripts to be published regarding fertility intentions in Europe shows that fertility plans changed for women in Germany and France, especially through postponement, while in Italy, the proportion of forgone is much higher than in the other countries, especially among younger and low-educated individuals (Luppi; Arpino; Rosina, 2020). The results for these European countries are also stratified. Higher education level is associated with postponement in Spain, whereas in Germany and France, it is associated with forgoing (Luppi; Arpino; Rosina, 2020). The same authors later published another study about Italians’ fertility plans in 2020. They found people in vulnerable occupations show a lower probability of intending to have a(nother) child and a higher probability of abandoning their pre-COVID fertility plan in the short term. Those who had income losses and with negative expectations about income and occupation were more likely to abandon their pre-pandemic fertility plan in the short and long term (Arpino; Luppi; Rosina, 2021).

A study in the U.K. found that only 9% of respondents (n=789) reported a change in fertility intention after the COVID-19 pandemic. Increased financial insecurity was predictive of changing intentions downwards (Raybould; Mynarska; Sear, 2023). In Poland, two studies also focused on occupation (Malicka; Mynarska; Swiderska, 2021; Kurowska; Matysiak; Osiewalska, 2023). The first study (Malicka; Mynarska; Swiderska, 2021) showed that almost 25% of the sample had intentions to have a child, but 20% of them had either postponed or forgone their fertility because of COVID-19. Financial insecurity is associated with postponing fertility at the full model, except when controlled by mental health. In this research, respondents could leave comments about how the pandemic had interfered with their childbearing intentions. The analysis of these qualitative materials shows how worsening material conditions, risks of unemployment, and an unstable financial situation were cited, as well as concerns for health and restrictions in health services (Malicka; Mynarska; Swiderska, 2021).

The second study for Poland (Kurowska; Matysiak; Osiewalska, 2023) evaluated fertility intentions associated with remote working and found that women who gained access to remote working had declining fertility plans. In cases where remote working was accompanied by worsened financial conditions, the chances of decreasing intention declined. This was also mediated by how egalitarian were the unions. Women who shared childcare with partners and worked remotely were less likely to increase fertility intentions than mothers who shared childcare but did not have access to remote work or women who did not share childcare before the pandemic. As mothers in egalitarian relationships working remotely were faced with paid and childcare work (which included homeschooling) at home, the extra burden contributed to decreasing fertility intentions (Kurowska; Matysiak; Osiewalska, 2023).

For Austria, less than 10% of people changed fertility plans due to the pandemic, with those who were already parents more likely to decline fertility intentions or postpone. Changes in fertility timing were more frequent than changes in quantum. In fact, childless and young adults did not change intentions because of the pandemic (Buber-Ennser; Setz; Riederer, 2024). They also discovered that older individuals are more likely than younger ones to revise their childbearing intentions negatively. The authors partially attribute this fact to the tendency of older individuals to be surrounded by peers who are also parents. Consequently, the challenges of balancing work and family life may be more apparent to older respondents, influencing their expectations about the implications of having children, especially during the pandemic.

A study for Australians (aged 18–45) used difference-in-difference models to compare changes in fertility intentions of the population who experienced a lockdown (located in the Victorian region) with the population from other areas that did not undergo a lockdown. They observed a small decline in reported intentions of having another child among women who lived through the lockdown, with more pronounced effects in older, less educated women, and those employed on fixed-term contracts (compared to unemployed or casual workers, who had a positive effect) (Mooi-Reci *et al*., 2022)

Three studies from the United States also show interesting results. In terms of socioeconomic effects, a study that used the Desire to Avoid Pregnancy (DAP) scale, a validated measure of pregnancy intention, found a decreased desire for 25% of respondents and no change for 34% of respondents. One-third of respondents felt scared to be pregnant and the decline in desire is associated with the inability to afford food, transportation, and/or housing (Lin *et al*., 2021). In another study of women actively trying to conceive, researchers found that one-third of participants changed preferences, with 23.9% anticipating fertility and 61.6% postponing fertility. Depressive and anxiety symptoms contributed to postponing fertility, as well as being older and having lower social support. Income does not make a difference, but having one child increases the odds of postponing compared to those who do not have any children. Having two or more children is not significant compared to zero children (Naya; Saxbe; Dunton, 2021). Another study for New York also found parity effects. Half of all women who had been trying to conceive or who were thinking about conceiving before COVID-19 stopped doing so in the first months of the pandemic. Once again, results were worse for women who were mothers of young children (Kahn *et al*., 2021).

Only one study has looked at fertility intentions during the pandemic in Brazil. Using panel data, Marteleto and colleagues examined the time-varying determinants of changing fertility intentions while accounting for unobserved, time-invariant individual factors using fixed effects models (Marteleto *et al*., 2023a). They find that high and/or increasing COVID-19 exposure at community level and perceived risk of COVID-19 infection at the individual level are associated with a greater likelihood of abandoning initial childbearing plans and a greater likelihood of maintaining intentions to forgo *versus* to intend to have additional children. Importantly, they advance the literature by highlighting how individual-level COVID-19 infection risk perceptions matter for fertility intentions, net of community-level exposure.

Yet, only a few of the studies discussed above, whether in Brazil or in other countries, examined the impact of pandemic-induced income loss directly on fertility intentions.

## Materials and methods

### Data

Between May and September 2020, the DeCodE Project (Demographic Consequences of Epidemics) conducted 25-minute phone interviews with 3,996 women aged 18–34 in Pernambuco, Brazil, the state most affected by Zika (Table A1). Respondents were recruited using a Random Digit Dialing technique through Computer Assisted Telephone Interviewing. To recruit a probabilistic sample, we used a list of randomly-generated cell phone numbers from Brazil’s government concession with more than 19 million numbers. Following convention, we examined partnered women (Hayford; Agadjanian, 2019), but extended the literature by including women in both formal and informal unions. Our analytic sample includes 1,524 partnered women;^3^ 26.3% have no children, 34.0% have one child and 37.7% have two or more children, in line with Pernambuco’s low fertility rate (1.67 TFR) (IBGE, 2022).

Qualitative data come from 56 semi-structured interviews conducted privately via Zoom/WhatsApp video in April-May 2020 (Table A2), that is, during the emerging months of the pandemic in Brazil. This is a key point. While the 7-day moving average of deaths in the country was low, still below a thousand per day (Worldometer, 2024), the uncertainty of what was coming was at its highest point.

Three experienced local team members used snowball sampling recruitment to search and find women from different education levels, and whether they had a pregnancy or birth during the Zika epidemic in Recife, the capital of Pernambuco (Table A2). To be eligible to participate, women needed to be between 18 and 34 years old and could not be included in the longitudinal sample. The first and last authors, along with a fieldwork coordinator, trained local female PhD candidates with experience in qualitative data to conduct the semi-interviews. Interviewers matched the racial profile of interviewees to reduce bias in responses. The instrument had questions on household unpaid work, childcare, financial situation, marital and sexual relationships, reproductive intentions, and pregnancy history, contingencies created by the pandemics, and fear of the pandemic, among many others. Recorded interviews averaged 68 minutes, and although most women were in their homes under lockdown, they were able to secure privacy from other family members during the interviews. Interviews were transcribed by trained graduate students and later deposited into an online qualitative research software called Dedoose to organize primary themes, highlight excerpts, and quantify codes and combinations (Corbin; Strauss, 1990; Creswell; Poth, 2016). Data collection had been approved by the Brazilian National Commission on Research Ethics (CONEP, CAAE: 34032920.1.0000.5149).

### Methods

We implemented multinomial logistic regression models. Our dependent variable was fertility intention at the onset of the pandemic. The comparison group was “intending pregnancy < 6 months” *versus* “postponing pregnancy for 7 >= months” and “forgoing childbearing altogether”. The comparison group was “intending pregnancy < 6 months” *versus* “postponing pregnancy for 7 >= months due to COVID-19” and “forgoing childbearing altogether due to COVID-19.” In sensitivity analysis, we coded postponement in additional ways – within the next year and the next two years. We present models on intentions within the next six months.

The focal independent variables were whether the household experienced income loss in the past four weeks (yes/no), and parity, coded as two options: motherhood status (yes/no) and number of children (none, one, two or more). The first option was used in the initial set of models to estimate relative risk ratios, while the second option was used to interact with income loss to generate predicted probabilities. We controlled for race (white, *parda*, black), age, age squared, education (high school or lower *versus* some college or higher), household income (< 1, 1–2, 2–3, 3 + minimum wages), marital union (yes/no), and whether the respondent reached ideal family size (fewer, ideal, more).

We implemented three sets of nested models: 1) all controls and motherhood/parity (yes/no); 2) added income loss; and 3) added an interaction term between motherhood/parity and income loss. We estimated the predicted probabilities at varying levels of the interaction term with all controls at their mean based on Model 3 with parity coded as 0, 1, and 2 + using Stata 16.

As for the qualitative data analysis, to ensure consistency, the coding process demanded a strict written manual built mainly deductively, but also inductively. The first codes and themes were listed based on the literature on the consequences of exogenous crises in the household, including reproductive consequences. As the team proceeded to read and code the entire material, a few new codes emerged from the data and were added to the codebook, followed by the re-verification of previous transcripts using an iterative approach (Coffey; Atkinson, 1996; Weiss, 1994). To guarantee consistency, every transcription was reviewed sequentially by the researchers. When a mismatch was found, codes were discussed in a weekly meeting until a consensus or a new code was created. Finally, all codes were organized into themes and thematic networks following the methodology used by Attride-Stirling (2001). All authors organized the selected quotes by parity to evaluate heterogeneous experiences, check whether saturation was met, and discuss discordant findings.

## Results

### Fertility intentions at the onset of the pandemic

**Figure 1.**
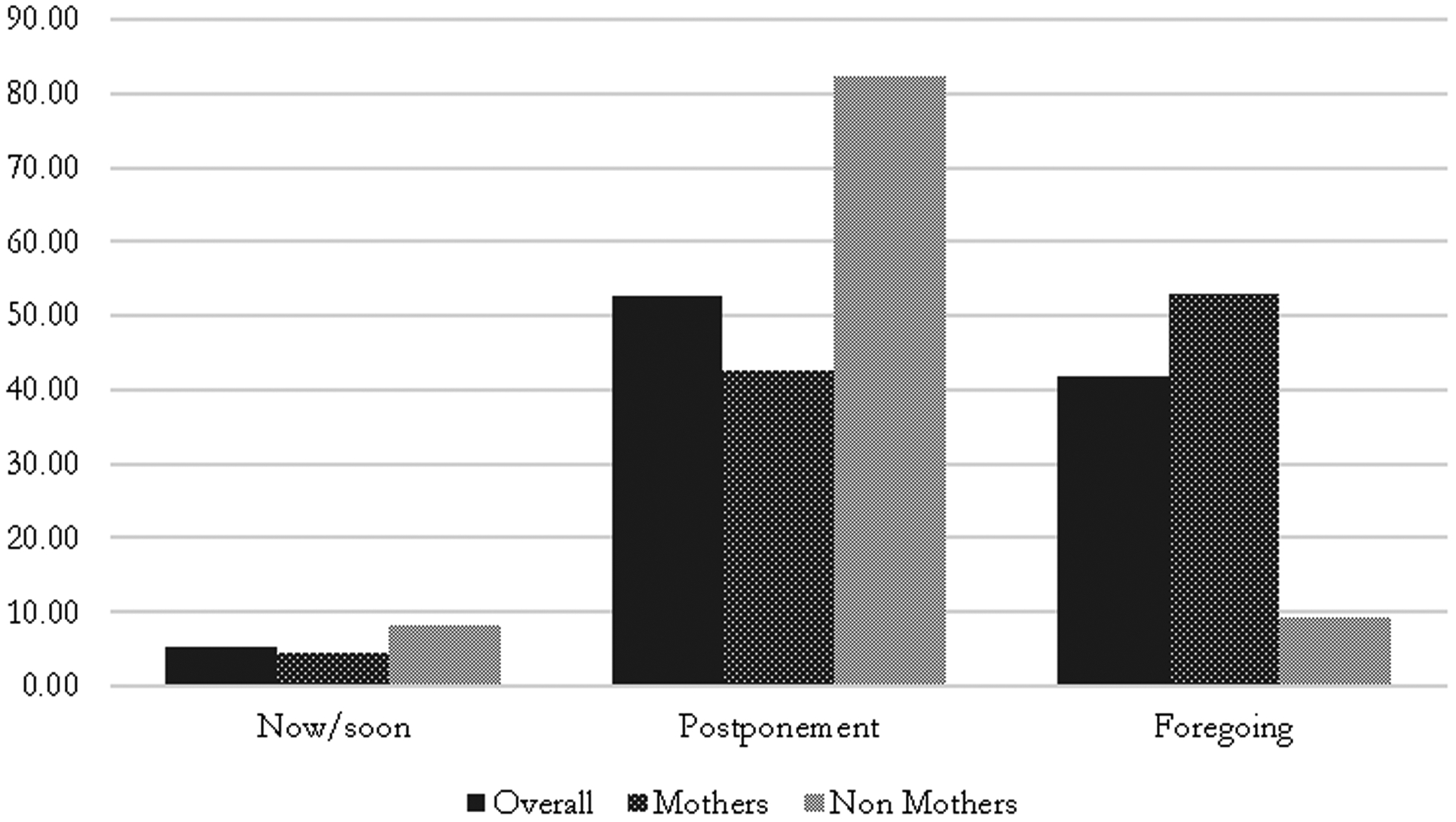
Fertility Intentions of Women Age 18–34 during the Covid-19 Pandemic by Motherhood Status: 2020 [N=1,524] Source: DeCoDE 2020.

Table 1 shows that most women wanted to avoid a pregnancy for at least six months at the onset of the pandemic: 51.4% (postponement), 43.9% (forgo), and 4.7% (within 6 months). Figure 1 shows fertility intentions for mothers and non-mothers separately. Combined, 4.4% of mothers intended a pregnancy within six months, compared to 8.3% of non-mothers. A greater proportion of non-mothers intended to postpone a pregnancy (82.4%) compared to mothers (42.6%). A larger proportion of mothers (53.0%) than non-mothers (9.3%) intended to forgo a pregnancy altogether.

This highlights two dimensions of differences by motherhood status. First, non-mothers were slightly more likely than mothers to intend a pregnancy soon, but an even greater proportion of non-mothers intended to postpone a pregnancy compared to mothers. Second, intentions to avoid pregnancy manifested differently for mothers and non-mothers. For most mothers, intentions to avoid pregnancy meant forgoing childbearing altogether, whereas for most non-mothers, intentions to avoid pregnancy meant postponement.

### Pandemic-induced income loss

Table 1 also shows that 47.3% of respondents experienced income loss early in the pandemic. The semi-structured interviews provide a more nuanced account of these experiences during this uncertain period. Women reported multiple ways in which they experienced income loss – losing a job, informal work or reduced working hours, and price increases. Márcia, a mother of two children stated: “There is [financial difficulty], because I’m not working anymore and my husband has a snack stand in a school. But schools aren’t open. So, we had to close, right?” Luana, a respondent with no children, said: “So, we lost the opportunity to work, because nobody here is a formal employee, we don’t have a job, everybody is self-employed. We used to get out to do one activity or another [informal work] but we can’t do that [due to lockdown].”

Respondents talked about how pandemic-induced economic conditions negatively impacted their ability to buy food. Several mothers of school-age children related how much more were their children eating at home. This is important given that with pandemic-related school closures, students lost access to government-subsidized school meals.

### Income loss, parity & fertility intentions

Table 2 shows estimates of multinomial logistic regression models of fertility intentions at the onset of the pandemic. Mothers were significantly more likely than non-mothers to intend to forgo a pregnancy at the onset of the pandemic (Model 1b). The difference between mothers and non-mothers remained when controlling for income loss (Model 2b).

**Figure 2.**
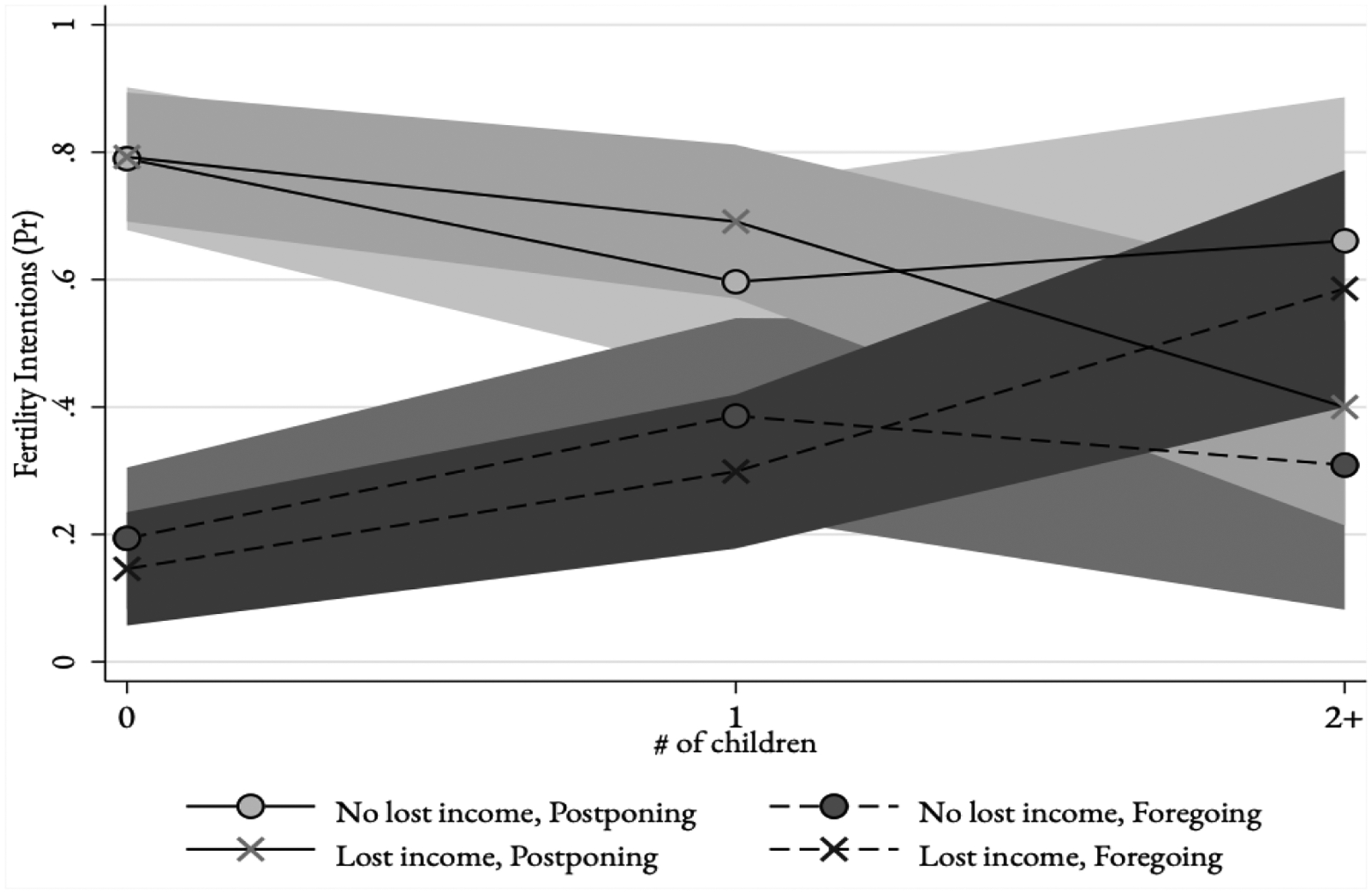
Predicted Probabilities of Fertility Intentions (Now, Postponing, Forgoing) at the Onset of the Pandemic by Parity and Income Loss, Pernambuco, Brazil, 2020 [N=1,524]. Source: DeCoDE 2020.

The next set of models shows how income loss and parity interacted to shape fertility intentions (Models 3a and 3b). Figure 2 reports the predicted probabilities based on these models and shows that both groups of mothers (1 and 2+ children) equally intended to forgo a pregnancy at the onset of the pandemic, compared to non-mothers. Non-mothers and mothers of one child who lost income intended to postpone or forgo pregnancy similarly to those who did not lose income. However, mothers of two or more who lost income in the early months of the pandemic differed significantly from those who did not lose income: those with income loss intended to forgo a pregnancy at higher rates than postpone a pregnancy, whereas those who did not experience income loss were more likely to intend to only postpone a pregnancy.

The semi-structured interviews corroborate these patterns, suggesting that mothers intended to avoid a pregnancy during the pandemic, citing hardship and instability due to pandemic-induced income loss as a principal motivation. For mothers of one child, the intention to avoid pregnancy during the pandemic was expressed as postponement. Respondents noted that postponing childbearing was related to experiences of pandemic-induced income loss, hardship, and economic instability. Zoe, a mother of one, stated: “So, [the pandemic] has changed everything. Absurdly. Because if I got pregnant now, it would be something very… I don’t even know. It would change our financial life completely. The Coronavirus is already changing [our financial life]. A child now would be perturbing. My friends who were planning a pregnancy now, to have a second child, they stopped with the plan you know? They will wait for the coronavirus to end.” Zelia, another mother of one, stated: “I wanted to be in a more stable financial situation [before a pregnancy] because of this pandemic; we are facing some money issues because of the pandemic… We couldn’t imagine… We are experiencing hardship right now and since we waited this long for another child, we will wait more so as not to get more [troubled] with bringing a child in the world, more than we already are with money now.”

Similar to mothers, non-mothers were also concerned about income loss and economic instability during the pandemic, and the difficulties associated with a potential pregnancy. However, non-mothers seemed to equalize pandemic-induced financial instability with other dimensions of uncertainty inherent to childbearing and life in general. Non-mothers were ambivalent about their fertility intentions in the face of economic uncertainty, leaving room for a pregnancy despite pandemic-induced income loss. Elaine, a respondent with no children, stated:“I believe I don’t know whether I would like to have a child now, because I can’t be really sure… the issue is… security. I mean security regarding all aspects, like, particularly security [in knowing] that they would have access to things in general, because I don’t know if I have the financial condition to afford [a baby], you know? The current situation, including the political situation, doesn’t favor having a child for financial reasons, like, to want to provide a life….” Along the same lines, Gwen, a respondent with no children said: “Yes… the pandemic affects me a lot because of… going to the doctor… also because of financial issues, we don’t know what tomorrow will bring. But I have no idea how long this [pandemic] will last, I don’t know if, for example, I got pregnant today, how things will be in nine months, when I go into labor. But [the pandemic] hasn’t negatively influenced a great deal so that I would say: ‘no, I won’t get pregnant now’.”

Other non-mothers said that pandemic-induced income loss and economic instability did not affect their intentions directly. A frequent issue among non-mothers considering childbearing during the pandemic was the uncertainty of how long the pandemic would last. Non-mothers discussed how, as pregnancy and childbearing are inherently uncertain situations, one more uncertain outcome did not seem to make much of a difference.

## Discussion

Combining analysis of population-level survey data and qualitative interviews, this study is among the first to disentangle how fertility intentions are associated with pandemic-related income loss and whether these associations vary by motherhood status. Our study contributes three main findings that advance understanding of the demographic repercussions of the pandemic. First, most women intended to avoid a pregnancy at the onset of the pandemic, and this was more frequent among women who experienced pandemic-induced income loss prior to the interview. Second, the meaning of avoidance differed between intentions to postpone *versus* forgo pregnancy. Third, non-mothers were more likely to intend postponing *versus* getting pregnant soon, whereas mothers were more likely to intend to forgo a pregnancy.

Broadly, our findings raise questions about how women can achieve their fertility intentions, whether they have more or fewer children than they intend or desire. The large proportion of unintended pregnancies in Brazil before the pandemic (Le *et al*., 2014; Theme-Filha *et al*., 2016) adds urgency to this question, as do the strains on Brazil’s healthcare system and access to reproductive health in particular, brought on by pandemic-related needs and policy changes (Diniz; Cabral, 2022; Oliveira *et al*., 2021). In Pernambuco, 82.6% of the population relies on the public healthcare system (IBGE, 2022). The interruption of contraceptive supply, health clinic closures, and shifting healthcare resources have heightened unmet need for healthcare (Oliveira *et al*., 2021) and declines in care quality, with reports of reproductive rights violations, obstetric violence, and maternal mortality (Diniz; Cabral, 2022).

More specifically, our findings indicate that intentions to avoid pregnancy at the onset of the pandemic had different meanings – postponing *versus* forgoing – depending on motherhood status, parity, and pandemic-induced income loss. Non-mothers were more likely to intend a pregnancy soon compared to mothers, and income loss did not make a difference. Mothers, on the other hand, had different intentions depending on income loss, with mothers losing income more likely to both postpone and forgo pregnancy (and to report intention change because of COVID-19), and mothers of two or more children experiencing income loss more likely to forgo pregnancy altogether.

Non-mothers articulated that income loss and instability, as well as uncertainty about the duration of the pandemic, were part of a number of uncertainties women already face regarding childbearing, and thus, having a child now *versus* later might not make much difference. This aligns with research showing that uncertainty, whether financial or not, is associated with desire for childbearing in some circumstances (Trinitapoli; Yeatman, 2018) with predictive power to short-term fertility outcomes (Yeatman; Trinitapoli; Garver, 2020).

Mothers, on the other hand, expressed a stronger intention of avoiding pregnancy during the pandemic, pointing to income loss and economic instability as reasons. This difference might be partly attributable to mothers’ financial and time burdens during the pandemic (e.g. childcare costs and responsibilities), which might make the additional constraints of another child seem too high. This is in line with research showing that childbearing ambivalence is a meaningful construct reflecting uncertainty associated with economic factors (Sennott; Yeatman, 2018). Another important reason might be that mothers are also more likely than non-mothers to be closer to their ideal family size already.

Overall, findings shed light on the connections between pandemic-induced income loss, motherhood, and fertility intentions. The severity of the pervasive pandemic-induced economic crisis suggests an enduring obstacle for women to meet their fertility intentions post-pandemic. Whether to remediate the consequences of the COVID-19 pandemic or of yet-to-come public health crises, findings from this study can help policymakers align their work to best serve all women. For example, practitioners might wish to engage women in conversations about their fertility intentions and their options for contraception if they intend to avoid pregnancy. Such conversations might benefit from an expanded discussion of contraception options, especially given reports of interruption of contraceptive access during the pandemic. At the same time, policymakers can plan and prepare social policies that safeguard reproductive plans during future economic and public health crises, such as financial subsidies for families with children or who are planning to conceive, acceleration of reopening of schools, and mandatory flexible work hours for parents during lockdowns or health emergencies.

Importantly for demographers, Brazil has observed large declines in fertility rates coupled with increasing mean age at childbearing in the last decades. Fertility levels have reached low levels and low fertility in Brazil does not seem to be short-lived or linked exclusively to public health and economic crisis, but a new reproductive profile in which women have few children, with many having fewer children than desired, a violation of reproductive rights. In this era of low fertility levels, it becomes even more pressing for demographers to study fertility preferences, paying particular attention to the factors that contribute to changes in this dynamic process.

It is worth noting that a crisis such as the pandemic can impact both immediate and long-term childbearing plans. We know that shocks can leave scars that might affect childbearing intentions and preferences throughout their life course. The concepts of social proximity to disease (Marteleto *et al*., 2023b) and demographic memory (Denton; Spencer, 2021) are important here. Individuals who know people who were infected or passed away during COVID-19 and are at the age of “remembering” this event may find that social proximity and demographic memory of a novel infectious disease crisis perpetuates its effect throughout people’s life courses and in a population. As such, a generation that spent a large part of its reproductive years battling repeated novel infectious disease crises and facing their negative consequences may be directly affected by that, not only in the short but also in the long term. For instance, the literature on fertility preferences has shown this to be the case for a new generation of youngsters in several European countries facing environmental crises.

To this end, findings from this study suggest that fertility preferences are sensitive to women’s immediate social and economic experiences, which are complex but, at the same time, also prone to improvements. It is time to discuss the conditions that would make fertility preferences less sensitive to shocks like the pandemic, such as children-friendly policies, subsidized and/or free full-time childcare, and integration of childcare in the workplace, among many others. Given Brazil’s current low fertility regime, it is time to recognize that we need to provide women with more than just access to healthcare and contraceptives; rather, to create conditions to help them cope with enduring instability, a marked characteristic of the second demographic transition. This will allow women and families to achieve their desired fertility preferences, whether in periods of high or low uncertainty.

## Figures and Tables

**Table 1.** Descriptive Statistics ~ Partnered Women Ages 18–34, Pernambuco, Brazil, 2020 [N=1,524]

Variable	Percent (%)
**Dependent Variables**	
Fertility Intentions	
*Now/Soon (within 6 mo.)*	4.7
*Postpone (7 mo. or more)*	51.4
*Forego*	43.9
Change in Fertility Intentions due to Covid-19	
*Now/Soon (within 6 mo.)*	12.4
*Postpone due to Covid-19*	64.0
*Foregoing due to Covid-19*	23.6
**Independent Variables**	
Childbearing	
*Non-mother*	26.3
*One child*	36.0
*2+ children*	37.7
Hh Income Loss	
*Yes*	47.3
*No*	52.7
Schooling	
*Incomplete college or more*	28.0
*High-School or less*	72.0
Race	
*White*	28.8
*Parda*	62.1
*Black*	9.1
Monthly Household Income	
*<1 MW*	40.9
*1–2 MW*	30.0
*2–3 MW*	13.1
*>3 MW*	16.1
Type of Union	
*Formal*	56.9
*Informal*	43.1
Ideal Family Size	
*Ideal*	26.7
*Fewer*	12.1
*More*	61.2
Age	
*Mean*	27.6
*N*	1,524

Source: DeCoDE 2020.

**Table 2.** Relative Risk Ratios from Multinomial Logistic Regressions for Pregnancy Intentions ~ Partnered Women Ages 18–34, Pernambuco, Brazil, 2020. [N=1,524]

	Model 1		Model 2		Model 3	
Variable	Post.	Forgo	Post.	Forgo	Post.	Forgo
	a	b	a	b	a	b
Parity (0/1+child)						
* ~Mother*	1.8+	5.3***	1.9+	5.3***	0.7	1.4
	(0.9,3.6)	(2.3,11.9)	(1.0,3.8)	(2.3,12.2)	(0.3,1.7)	(0.5,4.5)
Hh Income Loss			0.7	0.9	0.3**	0.2*
			(0.3,1.8)	(0.3,2.5)	(0.1,0.7)	(0.1,0.7)
1+child * HH Income Loss					5.8*	10.3**
					(1.4,23.6)	(2.0,52.0)
SES *~ High School*	0.6	1.1	0.6	1.1	0.6	1
	(0.3,1.5)	(0.4,2.9)	(0.3,1.5)	(0.5,2.8)	(0.3,1.3)	(0.4,2.5)
Race						
* ~Parda*	1.3	1.1	1.2	1.1	1.2	1
	(0.5,3.2)	(0.4,3.1)	(0.5,3.1)	(0.4,3.0)	(0.5,3.0)	(0.4,2.9)
* ~Black*	1.4	2.7	1.3	2.7	1.4	2.9
	(0.4,4.5)	(0.7,10.4)	(0.4,4.4)	(0.7,10.1)	(0.4,4.9)	(0.7,11.3)
Income						
* ~<1MW*	0.6	0.8	0.7	0.8	0.7	0.9
	(0.2,2.0)	(0.3,2.8)	(0.2,2.3)	(0.2,2.9)	(0.2,2.3)	(0.3,3.0)
* ~1–2MW*	1	1.6	1	1.6	1.1	1.7
	(0.3,3.6)	(0.4,6.1)	(0.3,3.6)	(0.4,5.9)	(0.3,3.8)	(0.5,6.3)
* ~2–3MW*	0.7	0.6	0.8	0.6	0.8	0.6
	(0.2,2.7)	(0.2,2.4)	(0.2,2.8)	(0.2,2.3)	(0.2,2.7)	(0.2,2.3)
Ideal Family size						
* ~Fewer*	1	1.1	0.9	1.1	1	1.1
	(0.1,8.8)	(0.2,7.8)	(0.1,8.3)	(0.2,7.6)	(0.1,8.8)	(0.2,7.9)
* ~More*	0.2+	0.0***	0.2+	0.0***	0.2+	0.0***
	(0.1,1.1)	(0.0,0.1)	(0.1,1.2)	(0.0,0.1)	(0.0,1.1)	(0.0,0.1)
Partner ~ *Informal*	1.2	1	1.2	1.1	1.3	1.2
	(0.4,3.3)	(0.4,3.0)	(0.5,3.2)	(0.4,3.1)	(0.5,3.4)	(0.5,3.4)
Age	0.5	0.8	0.5	0.8	0.5	0.8
	(0.1,1.8)	(0.2,3.8)	(0.2,1.8)	(0.2,3.7)	(0.2,1.8)	(0.2,3.6)
Age^2	1	1	1	1	1	1
	(0.1,1.1)	(0.0,0.1)	(0.1,1.2)	(0.0,0.1)	(0.0,1.1)	(0.0,0.1)

Source: DeCoDE 2020.

*** p<0.001;

** p<0.01;

* p<0.05,

+ p<0.10.

95% CI in parentheses. Also controlling for month of interview.

## Footnotes

1 Research shows how cases and death rates were not uniformly distributed across municipalities and states in Brazil (Castro *et al*., 2021a). In general, losses in life expectancy in the Northeast region were less pronounced than all other macroregions in the country in the first year of the pandemic, and Pernambuco was among the states with smaller declines in life expectancies in the first year of the pandemic (Castro *et al*., 2021b). Other work that published adjusted rates for the state capitals (Silva; Jardim; Lotufo, 2021), found that Recife, the Capital of Pernambuco, presented the second highest rate of the Northeast (184.54/100,000 inhabitants), behind Fortaleza (191.75/100 thousand). But both were much smaller than Manaus (253.6/100 thousand) and Rio de Janeiro (253.2/100 thousand), the two most affected capitals.

2 For a review, see Leocadio, Verona and Wajnman (2023).

3 We excluded respondents with missing information in at least one variable. Chi square and mean comparison tests of independence show that the missing variables are at random.

## Appendix

**Table A1.** DeCoDE Wave 1 Questionnaire Topics and IRB Information.

DeCodE Wave 1 Questionnaire Topics	
**Introduction**	Explain study length and topic. Contact information.
**Consent Form**	Participant’s full consent for participation. Additional contact Information.
**Zika, Coronavirus, and Dengue**	Has participant ever had Zika/Dengue/Covid-19? If yes, confirmed-suspected? How many people does participant know who have had Zika/Dengue/Covid-19? How many household members do they suspect have had Zika/Dengue/Covid-19? Do they know of babies with microcephaly or CZS? If so, were they the respondent’s children or other children?
**Relationships, Pregnancy and Children**	Marital status. Age at first marital union. Current Pregnancy status and how many weeks along? Pregnancy history (ever). History of sterilization, histerectomy Pregnancy outcomes: dates of birth, live births, weight, age, sex, # of adopted children Desire for sterilization. Ever had sex. Contraceptive use. Type of contraception. Access to contraception. Desired contraception.
**Childbearing and perspectives**	Future pregnancy intentions: effect of Covid-19 on these decisions and certainty. Feelings about pregnancy in next 3 months Ideal number of children in entire life. Delayed a potential pregnancy due to fear of Zika/Covid-19
**Fatalism and Perceptions**	Level of confidence in contraceptive method. Ability to speak about condom use with partner? Can they protect themselves from Covid-19? Can they protect themselves from Dengue/Zika?
**Health**	Health status. Health insurance. Unmet need for health services during Covid-19 Household/domestic work status. Physical or psychological violence at home Difficulty with paying for bills? Access to food? Worries related to Zika/Covid-19 (self, household member, pregnancy during Zika/Covid-19) Perceptions of getting Zika/Covid-19/Dengue. Knowledge about Zika/Covid-19. Fertility attitudes during Zika/Covid-19
**Demographic Variables**	Education. Mother’s education. Issues with access to water. Municipality in 2015, 2016, 2017, 2020, 2021. Household income, occupation, employment status, race, # of members in household, religion
**IRB information**	Data collection was conducted under Institutional Review Board approval #2018-01-0055 from the PI’s institution and the Brazilian National Commission for Research Ethics (also known as CONEP, or Comissão Nacional de Ética em Pesquisa) study approval CAAE: 34032920.1.0000.5149. Interviewers were formally trained to respect the confidentiality of the respondents. For the survey, if the participant was unavailable or in a public place, interviewers asked if they would prefer to schedule an interview for a later time to ensure privacy. Approval of deidentified data use is closely monitored by the PI. All authors and team members have completed CITI training to ensure compliance with 45 CFR 46, Subpart A, “Federal Policy for the Protection of Human Subjects.”

**Table A2.** Characteristics of Qualitative Sample (N=56), Women Ages 18–39, Pernambuco, Brazil, 2020.

Characteristics	Educational level		Total
	High school or lower	College or higher	
Age group			
18 – 25	14	1	15
26 – 34	12	29	41
Total	26	30	56
Race			
White	5	16	21
Non-white	21	14	16
Total	26	30	56
Marital Status			
Single	25	15	40
Married	1	9	10
Informal Union	0	2	2
Divorced	0	4	4
Total	26	30	56
Birth during Zika epidemicepideepidemics			
No	12	17	29
Yes	14	13	27
Total	26	30	56
Number of children ever born			
0	7	11	18
1	10	12	22
2	5	6	11
3 +	4	1	5
Total	26	30	56
Intends to have (more) children			
No	13	9	22
Yes	7	12	19
Doesn’t know	6	9	15
Total	26	30	56
